# Dual-Pseudorabies Viral Tracing for Spinal Tyrosine Hydroxylase Interneurons Involved in Segmental Micturition Reflex Circuitry in Spinal Cord Injured Rats

**DOI:** 10.1089/neur.2021.0045

**Published:** 2021-12-22

**Authors:** Jaclyn H. DeFinis, Shaoping Hou

**Affiliations:** Marion Murray Spinal Cord Research Center, Department of Neurobiology & Anatomy, Drexel University College of Medicine, Philadelphia, Pennsylvania, USA.

**Keywords:** bladder, external urethral sphincter, spinal cord transection, tyrosine hydroxylase, trans-synaptic tracing

## Abstract

Traumatic spinal cord injury (SCI) often leads to urinary dysfunction. Although an involuntary micturition reflex can be established to elicit voiding with time, complications arise in the form of bladder hyper-reflexia and detrusor-sphincter dyssynergia that cause incontinence and inefficient expulsion of urine. To date, the neuronal mechanisms that underlie regulation of micturition after SCI are not well understood. We recently observed an increase of a population of tyrosine hydroxylase (TH)^+^ cells in the rat lumbosacral cord post-SCI, which contribute to the sustention of a low level of dopamine that modulates the recovered bladder reflex. To identify whether spinal TH^+^ cells are involved in the micturition reflex pathway post-SCI, two isoforms of the trans-synaptic retrograde tracer, pseudorabies virus encoding green fluorescent protein (GFP; PRV-152) or red fluorescent protein (RFP; PRV-614), were injected into the bladder detrusor or the external urethral sphincter (EUS), respectively, 3 weeks after a spinal cord transection at the 10th thoracic level (T10) in rats. Immunohistochemistry was performed to examine infected TH^+^ cells in the caudal cord at both 48 and 72 h post-injection. As a result, double-labeled TH^+^/GFP^+^ and TH^+^/RFP^+^ cells could be found in the superficial dorsal horn, parasympathetic nuclei, and dorsal gray commissure (lamina X) at both time points. More importantly, a shared population of TH^+^ interneurons (TH^+^/GFP^+^/RFP^+^) exists between bladder and EUS circuitry. These results suggest that spinal TH^+^ interneurons may coordinate activity of the bladder and EUS that occurs during micturition reflexes post-SCI.

## Introduction

The lower urinary tract (LUT) has two main functions: periodic elimination and storage of urine. These two processes are dependent upon coordinated activity between the bladder smooth detrusor muscle and the striated external urethral sphincter (EUS). Coordination of these two muscles is achieved by reflex pathways in the spinal cord, brainstem, and higher brain centers.^1^ Specifically, the main spinal circuits controlling bladder and EUS activity are located in lumbosacral spinal segments.^2^ In normal conditions, this synergy is accomplished through a complex neural control system involving multiple neurotransmitters and neuropeptides at both central and peripheral levels.^3^ Among the many neuromodulators that regulate urinary activity, the dopaminergic (DA-ergic) system is closely linked to bladder pathology that manifests, for example, in Parkinson's disease wherein the damage of midbrain dopamine (DA) neurons causes micturition dysfunction.^4^ Additionally, several studies have revealed that neurons in the rat lower spinal cord express DA-ergic receptors, indicating that DA released within the spinal cord has the ability to regulate pelvic visceral function.^5–7^

Traditionally, it has been thought that the A11 diencephalospinal pathway is the sole source of DA to the spinal cord.^8^ However, work by Mouchet and colleagues revealed that tyrosine hydroxylase (TH)^+^ cells could be found in the rat spinal cord.^9^ In line with this, we recently observed a subpopulation of TH^+^ interneurons in the rat lower lumbosacral spinal cord that are distributed in autonomic regions.^6^ The location of these cells strongly suggests their involvement in pelvic visceral function, such as micturition. Interruption of supraspinal micturition pathways initially causes the bladder to become acutely areflexic.^10^ However, over a few weeks, reorganization of this circuitry ensues and there is partial recovery of urinary function through involuntary bladder and urethral reflexes.^11,12^ Notably, injury-induced plasticity of spinal TH^+^ cells coincides with the appearance of the recovered bladder reflex. This suggests that spinal TH^+^ cells could be a vital portion of the recovered urinary reflex after spinal cord injury (SCI).

Several studies have completed extensive tracing experiments of the LUT network in an effort to illustrate the complexity of this system.^13–15^ Pseudorabies virus (PRV) is transmitted from an infected neuron to other neurons through their synaptic connections, thus defining specific pathways involved in neuron-neuron signaling, including spinal interneurons.^16^ It has been assumed for many years that there is overlap in the spinal circuitry that controls the bladder and EUS, which stems from similarities in their labeling patterns when a tracer is injected into these organs individually.^13,14^ However, it remains unknown whether there are shared populations of interneurons that coordinate EUS and bladder function. This was largely attributable to the fact that, until the early 2000s, there were no trans-synaptic tracers that could be detected independently of each other.^17^ In other words, no such isoforms existed that could be utilized in the same animal. With the development of a new PRV reporter, however, came the ability to implement dual labeling of the EUS and bladder detrusor within the same subject.^18^

We previously demonstrated that TH^+^ interneurons are synaptically linked to the bladder detrusor neuronal network post-SCI.^6^ Further, pharmacological activation or blockage of spinal DA-ergic receptors altered the recovered EUS reflex, indicating that spinal endogenous DA, which is released by TH^+^ interneurons, regulates sphincter function.^19^ To determine the possibility that these cells are also involved in the coordination of bladder and EUS activity, we conducted dual pseudorabies viral tracing of the bladder and EUS within the same rat. The results of this study discerned whether spinal TH^+^ cells are a shared population of interneurons that modulate both LUT organs within the lumbosacral cord after SCI.

## Methods

### Animals

Adult female Wistar rats (weighing 200–250 g) that received a complete spinal cord transection were used in this study. Institutional Animal Care and Use Committee and National Institutes of Health guidelines on animal care were strictly followed to minimize the number of animals used and any potential suffering. After viral inoculation and histological analysis, 1 rat in the 72-h group was found to have very few motoneurons and almost no interneurons labeled, indicating insufficient viral infection. The data from this rat was therefore excluded for further statistics. A total of 12 rats were used for final analysis.

### Spinal cord surgery

To determine whether spinal TH^+^ interneurons are involved in recovered micturition reflex circuitry, we performed a complete spinal cord transection at the 10th thoracic level (T10-TX) to completely remove supraspinal control as well as some propriospinal projections onto spinal micturition neuronal circuits. Animals were anesthetized with 2% isoflurane. A partial laminectomy was performed at the T8 vertebrae to expose the dorsal spinal cord. The spinal cord was completely transected at T10 using a No. 11 blade. Lesion completeness was verified visually at the time of surgery. Overlying musculature and skin were then closed. Animals were administered lactated Ringer's solution (Baxter Healthcare, Deerfield, IL), cefazolin (10 mg/kg), and burprenex (0.1 mg/kg; Reckitt Benckiser, Slough, UK) post-operatively. Bladders were manually expressed two to three times daily until euthanized.

### Dual pseudorabies virus trans-synaptic tracing

The two viruses used in these experiments were PRV-152 and PRV-614 encoding green (GFP) or red fluorescent protein (RFP; both 10^9^ pfu/mL; Bartha strains, courtesy of Dr. Michael Lane), respectively. Three weeks after SCI, rats were anesthetized with 2% isoflurane. An abdominal incision was made to expose the urinary bladder. A portion of the pubic symphysis was removed to expose the EUS. A total of 6 μL of PRV-152 was slowly injected into the ventral bladder detrusor muscle and 6 μL of PRV-614 into the EUS with a Hamilton syringe fitted with a glass capillary tip (three sites, 2 μL per site).^18^ Injection sites were immediately sealed with a small drop of tissue glue. The incision was closed with sutures. Animals were perfused 48 or 72 h (both *n* = 6) post-injection for histological analyses.

### Tissue processing and immunohistochemistry

Rats were anesthetized and transcardially perfused with 0.1 M of phosphate-buffered saline (PBS; pH 7.4), followed by 4% paraformaldehyde in PBS. The spinal cord was dissected out and put in the same solution for post-fixation overnight. Tissue was then transferred to 30% sucrose in 0.1 M of Tris-buffered saline (TBS) for at least 24 h. One 3-cm lumbosacral spinal segment was embedded in gum tragacanth (Sigma-Aldrich, St. Louis, MO) in 30% sucrose/PBS for sectioning.^20^ Spinal cords were serially cryosectioned in the transverse plane at 20 μm in 10 series of mounted sections. Thus, spinal cord transverse sections 200 μm apart were used in each immunostaining process.^21^

For immunostaining, mounted spinal sections were incubated with primary antibodies diluted in blocking solution (TBS containing 5% donkey serum and 0.5% Triton X-100) overnight at 4^°^C. We used primary antibodies against TH (rabbit, 0.1 μg/mL; Millipore, Billerica, MA), RFP (mouse, 0.2 μg/mL; Rockland Immunochemicals, Inc. Limerick, PA), GFP (chicken 0.1 μg/mL; Abcam, Cambridge, MA), and choline acetyltransferase (ChAT; goat, 5 μg/mL; Millipore). Sections from both 48- and 72-h time points were double or triple stained for TH, GFP, and RFP to examine whether spinal TH^+^ interneurons are a part of LUT circuitry. Because some RFP^+^ cells were found within the lumbosacral parasympathetic nuclei (PN), immunostaining for ChAT, in combination with RFP and GFP, was performed to identify whether these cells were parasympathetic pre-ganglionic neurons (PPNs). After rinsing, sections were incubated in Alexa-488, -594, or -647 conjugated donkey secondary antibodies (1:500; Invitrogen, Carlsbad, CA) for 3 h at room temperature. Slides were cover-slipped with FluorSave mounting medium (Calbiochem, San Diego, CA). Sections were imaged using a Leica DM5500B microscope (Leica Microsystems, Wetzlar, Germany).

### Quantitative analysis

Quantification was conducted by an observer blind to group identity. A series of transverse L6/S1 spinal cord sections immunostained for TH, GFP, and RFP were used to quantify PRV-152 and -614 infected spinal TH^+^ interneurons. Total numbers of TH^+^ cells, TH^+^/GFP^+^, TH^+^/RFP^+^, and TH^+^/GFP^+^/RFP^+^ were counted in ∼10 sections per rat wherein PRV-labeled cells were detected. This analysis was completed in three areas of the spinal cord that are known to contain TH^+^ cells and be involved in pelvic visceral function: the PN, superficial dorsal horn (DH), and dorsal gray commissure (DGC).^6^ In each area, GFP- or RFP-labeled TH^+^ cells were added together in these sections as a final sample number for statistics.

### Statistical analysis

An unpaired Student's *t*-test was used to analyze these data. Statistical analyses were performed in GraphPad Prism software (version 9; GraphPad Software Inc., La Jolla, CA). Significance throughout all experiments was set at *p* < 0.05. Data are represented as mean ± standard error of the mean.

## Results

**FIG. 1.**
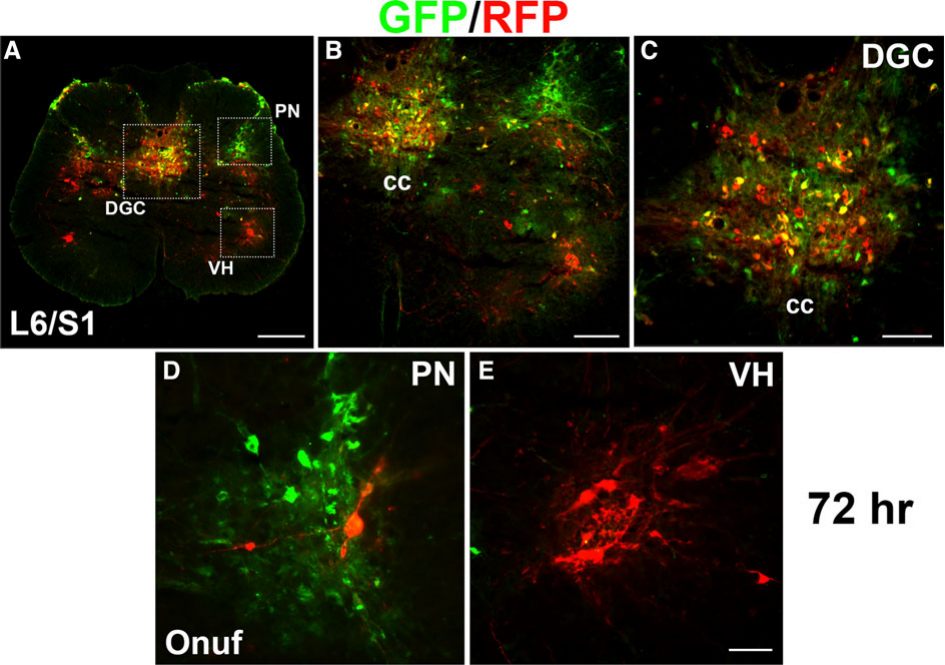
Dual PRV injections label interneurons in the lumbosacral cord after SCI. PRV-152 or -614 was injected into the bladder detrusor and EUS muscle 3 weeks after a T10 spinal cord transection. At 72 h post-inoculation, many GFP/RFP double-labeled cells are detected in the dorsal gray commissure (DGC), parasympathetic nuclei (PN), and superficial dorsal horn (DH) of the L6/S1 spinal cord (**A**–**C**). Extensive labeling of GFP^+^ neurons in the PN (**D**) and RFP-labeled neurons in Onuf's nuclei of the VH (**E**) can be seen. Scale bars: A, 200 μm; B, 100 μm; C, 50 μm; E, 35 μm. EUS, external urethral sphincter; GFP, green fluorescent protein; PRV, pseudorabies virus; RFP, red fluorescent protein; SCI, spinal cord injury; VH, ventral horn.

Because all animals were injected with both strains of PRV-152 and -614, we were able to examine TH^+^ interneurons that are engaged in bladder and EUS circuitry together regarding their connection with other populations of interneurons. After 48 h, injections of PRV-152 into the bladder detrusor mainly labeled neurons in the PN in addition to several labeled cells in the DGC and superficial DH of the L6/S1 spinal cord, whereas injections of PRV-614 into the EUS muscle infected motoneurons in Onuf's nuclei and a few in the PN, DGC, and DH. Some GFP/RFP double-labeled cells were observed in the PN, DGC, and DH. At 72 h post-inoculation, several single- or double-labeled cells were detected in these regions, particularly in the DGC (Fig. 1).

**FIG. 2.**
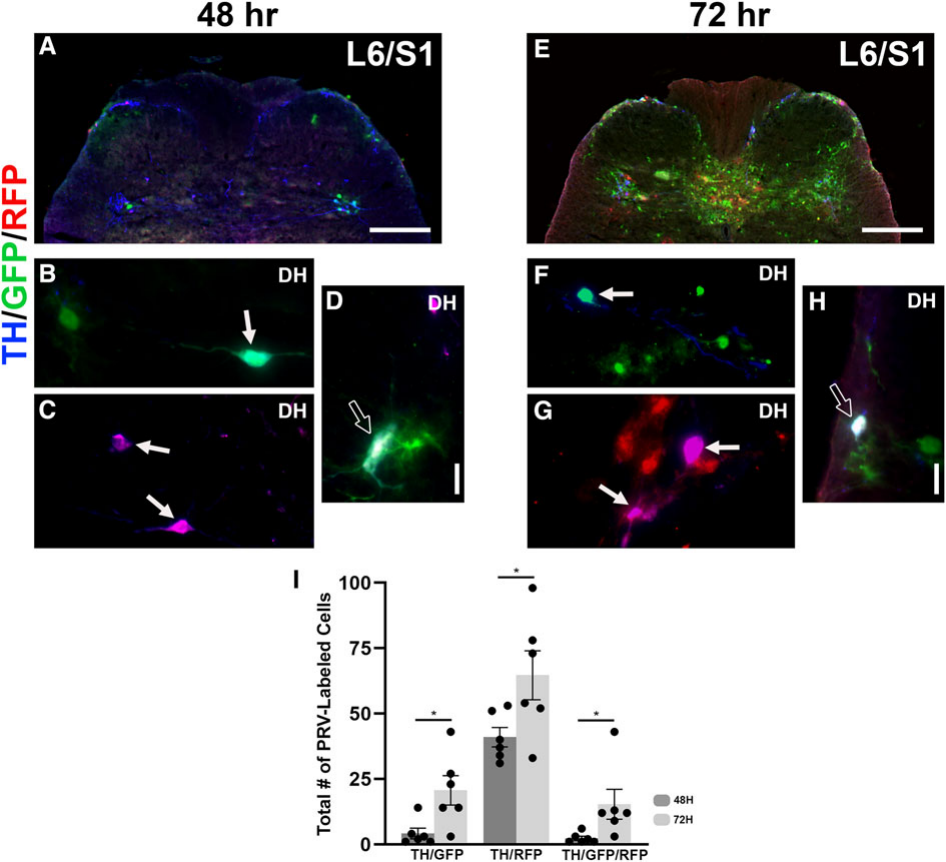
Spinal TH^+^ cells are synaptically linked to recovered bladder and sphincter reflexes. After injection of PRV-152 or -614 into the bladder detrusor and EUS muscle in SCI rats, double-labeled TH^+^/GFP^+^ or TH^+^/RFP^+^ cells can be found in the dorsal gray commissure (DGC), parasympathetic nuclei (PN), and superficial dorsal horn (DH) at both 48 (**A–C**) and 72 h (**E–G**) post-injection. Importantly, TH^+^/GFP^+^/RFP^+^ triple-labeled cells could be found in these regions. Representative images illustrate double- (white arrows) or triple-labeled (stroked arrows) cells in the DH at both time points (**D,H**). Statistical analysis shows that the number of double- and triple-labeled TH^+^ cells are greater at 72 versus 48 h post-injection (all *p* < 0.05, panel **I**). Scale bars: A,E, 200 μm; D,H, 20 μm. EUS, external urethral sphincter; GFP, green fluorescent protein; PRV, pseudorabies virus; RFP, red fluorescent protein; SCI, spinal cord injury.

Triple immunostaining for TH/GFP/RFP revealed that TH^+^/GFP^+^ double-labeled neurons reside in the PN, superficial DH, and DGC of SCI rats 48 h after injections. At 72 h, a similar pattern of labeled neurons was observed in the previously mentioned areas, with particularly strong expression in the DGC (Fig. 2A–C). Statistical analysis shows that the number of double- and triple-labeled TH^+^ cells was greater at 72 versus 48 h post-injection (all *p* < 0.05, unpaired *t*-test; Fig. 2I). When comparing the amount of TH^+^/GFP^+^ neurons in each area of interest between the 48- and 72-h cohorts, there were significantly more double labeled neurons in the superficial DH at 72 h (*p* < 0.01; Table 1), consistent with our previous reports that examined the extent of TH^+^/GFP^+^ detrusor circuitry labeling.^6^ After injection of PRV-RFP into the EUS, TH^+^/RFP^+^ double-labeled neurons were found in the PN, superficial DH, and DGC of the L6/S1 spinal cord at both 48 and 72 h (Fig. 2E–G). Importantly, there were significantly more double-labeled cells in the DGC at 72 h (*p* < 0.01; Table 1). At both 48 and 72 h post-injection, TH/GFP/RFP triple-labeled neurons were found in the PN, superficial DH, and DGC (Fig. 2A,D,E,H). Specifically, there were significantly more triple-labeled neurons in the superficial DH at 72 h (*p* < 0.05; Table 1). The results indicate that spinal TH^+^ interneurons are, in fact, involved in re-established bladder and EUS reflex circuitry post-SCI.

**Table 1.** The Number of PRV-Labeled Cells in the L6/S1 Spinal Cord

	Region	TH^+^/GFP^+^	TH^+^/RFP^+^	TH^+^/GFP^+^/RFP^+^
48 h	PN	3.83 ± 1.90	16.83 ± 1.76	1.83 ± 0.60
	DH	0.17 ± 0.17	20.33 ± 2.53	0.33 ± 0.33
	DGC	0.17 ± 0.17	3.83 ± 0.75	0.17 ± 0.17
72 h	PN	11.17 ± 2.93	24.17 ± 5.52	6.67 ± 2.26
	DH	7.33 ± 2.06^**^	25.83 ± 2.86	6.83 ± 2.15^*^
	DGC	2.17 ± 1.58	14.67 ± 3.12^**^	1.83 ± 1.44

^*^ *p* < 0.05; ^**^p < 0.01.

PRV, pseudorabies virus; PN, parasympathetic nuclei; DH, superficial dorsal horn; DGC, dorsal gray commissure; TH, tyrosine hydroxylase; GFP, green fluorescent protein; RFP, red fluorescent protein.

**FIG. 3.**
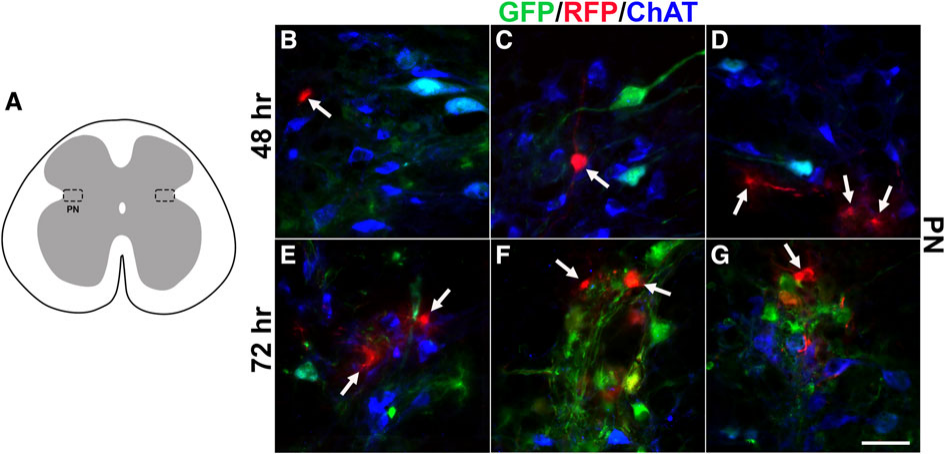
The majority of RFP-labeled cells in the parasympathetic nuclei are interneurons. To determine whether RFP^+^ cells in the PN are PPNs or interneurons, triple staining for ChAT, RFP, and GFP was conducted in the L6/S1 spinal cord. At both 48 (**A–C**) and 72 h (**D–F**), most GFP-labeled cells are ChAT^+^ in the PN, whereas the majority of RFP^+^ cells are ChAT^–^ and much smaller in size (white arrows), indicating that these cells are more than likely interneurons. Scale bars: 25 μm. ChAT, choline acetyltransferase; GFP, green fluorescent protein; PN, parasympathetic nuclei; PPNs, parasympathetic preganglionic neurons; RFP, red fluorescent protein.

Because RFP^+^ neurons were detected within the PN, where cholinergic parasympathetic PPNs are located, immunostaining for ChAT was also implemented to determine whether labeled cells were PPNs that appeared as a result of contamination of PRV-614 into the internal urethral sphincter (IUS). Results revealed that very few RFP^+^ neurons (less than two cells on average) in the PN were also cholinergic. Instead, most RFP-labeled cells within this area were, in fact, ChAT^–^ at both time points (Fig. 3). Notably, neurons that were RFP^+^/ChAT^–^ in the PN were noticeably smaller in size when compared to ChAT^+^ neurons that reside in the same area. The labeling pattern is similar to previous reports in which a very small number of ChAT^+^/RFP^+^ neurons were labeled in the PN when PRV was injected into the EUS.^14^ This suggests that the majority of RFP^+^ cells that are in close proximity to the PN are, in fact, interneurons as opposed to PPN efferents.

## Discussion

It was recently reported that lumbosacral TH^+^ interneurons are the sole source of DA in the spinal cord after a SCI interrupts the A11 diencephalospinal DA-ergic pathway.^6^ Here, we illustrated that this population of TH^+^ cells make synaptic connections with the LUT neuronal network. After SCI, both smooth bladder detrusor and striated EUS muscle reflex circuitry contain spinal TH^+^ cells within the superficial DH, PN, and DGC. This indicates that the two urinary organs share a population of interneurons that could serve to regulate the synergistic activity between the two muscles, which is an essential property for efficient voiding. Thus, injury-induced plasticity of TH^+^ interneurons is involved in the re-establishment of both bladder and sphincter segmental reflex pathways.

Spinal interneurons are crucial for the micturition reflex after SCI.^22, 23^ After the interruption of supraspinal pathways, a spinal micturition reflex develops because of intraspinal plasticity.^11^ This may be, in part, attributable to newly formed spinal circuits or the re-emergence of developmental reflex pathways.^24^ Based on our previous observations, lumbosacral TH^+^ interneurons seem to be a critical component of this recovered spinal reflex pathway that forms post-SCI.^6^ The injury-induced increase in the number of TH^+^ cells coincides with the partial recovery of bladder function that is observed subsequent to SCI. Depletion of these cells along with concomitant cystometry data have illustrated that, although not pertinent to micturition function in the adult naïve rat, TH^+^ cells play a much larger role in urinary function after SCI.^6^

Consistent with our previous findings, TH^+^ cells are involved in bladder detrusor muscle circuitry and double-labeled neurons reside in the PN. There were significantly more TH^+^/GFP^+^ neurons within the superficial DH at 72 h. In addition to validating earlier findings, we also sought to determine whether spinal TH^+^ cells were also synaptically linked to EUS function. With the use of PRV-614, which encodes an RFP reporter, we were able to detect TH^+^/RFP^+^ labeled neurons. Notably, there were significantly more double-labeled cells in the DGC 72 h post-injection.

The presence of TH/GFP/RFP indicates that there is a shared population of TH^+^ interneurons that innervate both the bladder and EUS.^18^ Although, this population of cells can be found in all areas of interest, the number of both TH^+^/GFP^+^ and TH^+^/GFP^+^/RFP^+^ neurons were significantly higher in the superficial DH at 72 h. Given that neurons in the DH are largely correlated with sensory input, this suggests that TH^+^ interneurons potentially play a role in sensory processing of the bladder and EUS. These results could imply that lumbosacral TH^+^ cells are extensively involved in the modulation of EUS function post-SCI. This indicates that these TH^+^ cells may play a role in integrating multiple reflexes concerned with micturition and speaks to the complexity of this system. These shared interneurons could have pre- and post-synaptic influences on pre-ganglionic motor neurons in the PN and on motoneurons in Onuf's nucleus in the ventral horn. When voiding is initiated in neuroaxis intact subjects, two crucial events occur: 1) the pontine micturition center within the rostral brainstem directly activates autonomic motoneurons within the sacral spinal cord and, at the same time, 2) it indirectly inhibits EUS motoneurons through gamma-aminobutyric acidergic and glycinergic interneurons.^25^ This allows for the simultaneous contraction of the detrusor muscle and relaxation of the EUS so that urine can flow through the urethra until the bladder is emptied.

Given that descending supraspinal control is disrupted post-SCI, this leaves only spinal regulation of both the bladder and EUS. A likely candidate for such modulation after SCI is spinal interneurons, which are synaptically linked to detrusor and EUS circuitry. Spinal TH^+^ neurons contribute to endogenous DA release in the injured spinal cord. According to our recent findings, spinal DA-ergic mechanisms regulate the micturition reflex in SCI rats. Specifically, tonically active D_1_-like receptors in SCI rats inhibit urine storage and enhance voiding by differentially modulating the EUS tonic and bursting patterns, respectively. On the other hand, pharmacological activation of D_2_-like receptors, which are normally silent, improves voiding by increasing EUS bursting. Enhancing DA signaling with the DA precursor, levodopa, or the non-specific DA receptor agonist, apomorphine, achieves better detrusor-sphincter coordination to facilitate micturition function in SCI rats.^19^ This indicates that spinal DA-ergic mechanisms play an important role in recovered micturition function and may serve as a novel therapeutic target after SCI. Thus, spinal TH^+^ interneurons emerge through an injury-induced response to compensate for the loss of descending control in an attempt to modulate the recovered bladder reflex in SCI rats.

Given that we found TH^+^/RFP^+^ neurons in the PN, there was concern that potential leakage of tracer into smooth muscle had occurred because of the close proximity between the EUS and IUS. However, immunolabeling for ChAT and RFP demonstrated that the majority of RFP^+^ cells were not ChAT^+^. This illustrates that specific labeling of smooth and striated muscle was achieved without significant overlap. It is also important to note that we focused solely on the injured spinal cord for these tracing studies because, compared to the injured spinal cord, the number of TH^+^ interneurons in the intact cord is much lower and they are not postulated to play a significant role in the regulation of micturition. This is likely due to the fact that supraspinal dopaminergic pathways, as well as others, that regulate voluntary control of voiding are intact. The detrusor and EUS muscle are located at such a distance that makes the two organs easily distinguishable when injecting. Though we did not examine the local expression of PRV in the bladder and EUS, a great number of infected neurons in the spinal cord indicates the high efficiency of viral injections. Otherwise, the distinct labeling pattern would not occur. Additionally, future studies will be performed utilizing a clinically relevant contusion injury model to assess the extent of TH^+^ interneuron regulation within micturition reflex circuitry.

## Conclusion

After trans-synaptic tracing of both the smooth bladder detrusor and striated EUS muscle, it was confirmed that spinal TH^+^ interneurons are synaptically linked to micturition reflex circuitry after SCI. This suggests that this subpopulation of DA-ergic neurons undergoes injury-induced plasticity to compensate for the loss of supraspinal DA supply in an effort to modulate both bladder and EUS recovered function in SCI rats. Ultimately, these experiments shed light on a potentially novel therapeutic target (e.g., spinal TH^+^ interneurons) for urinary dysfunction in SCI patients.

## Abbreviations Used

ChAT: choline acetyltransferase
DA: dopamine
DA-ergic: dopaminergic
DGC: dorsal gray commissure
DH: dorsal horn
EUS: external urethral sphincter
GFP: green fluorescent protein
IUS: internal urethral sphincter
LUT: lower urinary tract
PBS: phosphate-buffered saline
PN: parasympathetic nuclei
PPN: parasympathetic preganglionic neuron
PRV: pseudorabies virus
RFP: red fluorescent protein
SCI: spinal cord injury
TBS: Tris-buffered saline
